# Prostate Cancer Screening Uptake in Transgender Women

**DOI:** 10.1001/jamanetworkopen.2023.56088

**Published:** 2024-02-14

**Authors:** Sandhya Kalavacherla, Paul Riviere, Sruthi Kalavacherla, Jennifer T. Anger, James D. Murphy, Brent S. Rose

**Affiliations:** 1 Department of Radiation Medicine and Applied Sciences, University of California, San Diego, La Jolla; 2 Center for Health Equity, Education, and Research, University of California, San Diego, La Jolla; 3 Department of Biology, Massachusetts Institute of Technology, Cambridge; 4 Department of Urology, University of California, San Diego, La Jolla

## Abstract

**Importance:**

There is no consensus in prostate-specific antigen (PSA) screening guidelines regarding transgender women despite their known prostate cancer risk.

**Objective:**

To identify factors associated with recent (within the last 2 years) PSA screening in transgender women compared with cisgender men.

**Design, Setting, and Participants:**

This case-control study used data from the 2018 and 2020 Behavioral Risk Factor Surveillance System (BRFSS) surveys to characterize rates of PSA screening for prostate cancer within the past 2 years and multivariable logistic regressions to characterize factors associated with recent screening among transgender women. The BRFSS program of the Centers for Disease Control and Prevention annually surveys over 400 000 US adults on behavioral risk factors, chronic illnesses, and use of preventive services. Respondents to the BRFSS who were cisgender men or transgender women 40 years or older and who had complete PSA testing responses and no prostate cancer history were included; 313 transgender women and 138 937 cisgender men met inclusion criteria. Matching was performed by age, race and ethnicity, educational level, employment, annual income, survey year, and cost barriers to care. Data were collected on November 2, 2022, and analyzed from November 2, 2022, to December 3, 2023.

**Main Outcomes and Measures:**

Rates of and factors associated with recent PSA screening in transgender women.

**Results:**

Among the 1275 participants included in the matched cohort (255 transgender women and 1020 cisgender men; 570 [44.7%] aged 55-69 years), recent PSA screening rates among transgender women and cisgender men aged 55 to 69 were 22.2% (n = 26) and 36.3% (n = 165), respectively; among those 70 years and older, these rates were 41.8% (n = 26) and 40.2% (n = 98), respectively. In the matched cohort, transgender women had lower univariable odds of recent screening than cisgender men (odds ratio [OR], 0.65 [95% CI, 0.46-0.92]; *P* = .02). In a hierarchical regression analysis adding time since the last primary care visit, effect size and significance were unchanged (OR, 0.61 [95% CI, 0.42-0.87]; *P* = .007). After adding whether a clinician recommended a PSA test, there was no statistically significant difference in odds of screening between transgender women and cisgender men (OR, 0.83 [95% CI, 0.45-1.27]; *P* = .21). The results were further attenuated when clinician-led discussions of PSA screening advantages and disadvantages were added (OR, 0.87 [95% CI, 0.47-1.31]; *P* = .32). In a multivariable logistic regression among transgender women, having a recommendation for PSA testing was the factor with the strongest association with recent screening (OR, 12.40 [95% CI, 4.47-37.80]; *P* < .001).

**Conclusions and Relevance:**

In this case-control study of one of the largest cohorts of transgender women studied regarding PSA screening, the findings suggest that access to care or sociodemographic factors were not principal drivers of the screening differences between transgender women and cisgender men; rather, these data underscore the clinician’s role in influencing PSA screening among transgender women.

## Key Points

**Question:**

What factors are associated with recent prostate-specific antigen (PSA) screening in transgender women?

**Findings:**

In this case-control study of 255 transgender women and 1020 cisgender men matched by sociodemographic variables, transgender women had significantly lower odds of recent PSA screening than cisgender men when controlling for time since the last primary care visit. When clinician-led recommendations for and discussions of PSA screening were added, there was no longer a statistically significant difference between groups.

**Meaning:**

These data suggest that disparities in PSA screening among transgender women may be associated with clinician patterns of care rather than differences in sociodemographic characteristics or access to care.

## Teaser

This case-control study assesses use of prostate-specific antigen (PSA) screening in a matched cohort of transgender women and cisgender men using the Centers for Disease Control and Prevention Behavioral Risk Factor Surveillance System.

## Introduction

While prostate cancer is the second leading cause of cancer-related deaths in US cisgender men,^1^ limited literature is available on the incidence of prostate cancer and prostate-specific antigen (PSA) cancer screening among transgender women. Guidelines for PSA screening for cisgender men are age based, recommending that men aged 55 to 69 years discuss the benefits of PSA screening with a clinician and that men 70 years or older avoid PSA screening, as they are unlikely to benefit from early detection.^2^ There is no consensus, however, among cancer screening guidelines on PSA screening in transgender women, and most guidelines exclude transgender women entirely.^3^ These lack of guidelines may in part contribute to the lack of screening for prostate cancer in transgender women, who remain at risk for prostate cancer after transitioning, as prostatectomies are not performed during gender-affirming surgery due to the high complication risk.^4^

In addition to a lack of clinician awareness on transgender-specific issues, transgender populations face heightened barriers to health care stemming from discrimination and socioeconomic disparities.^5^ These barriers widen gaps in outcomes between transgender women and cisgender men and may have fatal consequences, as evidenced by the significantly lower uptake of many cancer screenings and treatment for transgender individuals.^6^ This trend is compounded by increased cancer morbidity and mortality among transgender patients; specific to prostate cancer, 1 study^5^ reported that transgender individuals experienced a significantly lower survival rate after diagnosis compared with cisgender counterparts. Given that transgender women may have worse prostate cancer outcomes and given the paucity of contemporary data on PSA screening for transgender women in the US, we studied use of PSA screening in a matched cohort of transgender women and cisgender men with data from the Centers for Disease Control and Prevention (CDC) Behavioral Risk Factor Surveillance System (BRFSS).

## Methods

This case-control study was exempt from approval by the University of California, San Diego, Health Institutional Review Board owing to the use of publicly available deidentified data. The study followed the American Association for Public Opinion Research (AAPOR) reporting guideline and the Strengthening the Reporting of Observational Studies in Epidemiology (STROBE) reporting guideline.

The BRFSS annually surveys over 400 000 randomly selected adults in the US via telephone on their behavioral risk factors, chronic illnesses, and use of preventive services, including PSA screening. The BRFSS survey response data are published online with weights that facilitate the generalizability of the conclusions made from the survey data to the national population.^7,8^ The BRFSS also publishes Summary Data Quality Reports to quantify participation rates and distributions of nonresponders.^9^

We selected and pooled data from the 2018 and 2020 BRFSS surveys, as these were the most recent years that included PSA questionnaires. Details about changes in state and nationwide BRFSS survey protocols in response to the 2020 shelter-in-place orders were published by the CDC.^10^ To identify transgender women, the cohort first included patients who indicated male sex assignment at birth. Transgender women were then defined as patients who answered the BRFSS survey question “Do you consider yourself to be transgender?” with “Yes, transgender, male-to-female.” Those who answered no to this question were considered cisgender men. We then analyzed demographic characteristics (age, race and ethnicity, annual income, educational level, and employment status) and access to care data, including time since last primary care visit for routine health maintenance and whether the respondent had a perceived cost barrier that prevented access to a clinician within the last 12 months. Race and ethnicity data were derived from either the patient-reported or imputed race (if the respondent refused to self-report, the value was the most common race response for that region). These data were collected because of previously described associations between race and utilization of preventive cancer screening resources in cisgender populations; we sought to identify whether such race-based disparities exist in the context of prostate cancer screening in transgender women. Further, responses to the following PSA-related questions were collected: “Have you ever had a PSA test?” (options: yes or no); “How long has it been since you had your PSA test?” (options: within the past year, 1-2 years ago, 2-3 years ago, 3-5 years ago, or >5 years ago [noninclusive upper bounds]); “Has a doctor, nurse, or other health professional ever talked with you about the benefits of the PSA test?” (options: yes or no); and “Has a doctor, nurse, or other health professional ever talked with you about the harms of the PSA test?” (options: yes or no). Respondents could select “don’t know/not sure” or refuse to answer any of these questions; these response types were recoded as not available within our analysis. Transgender women or cisgender men who had a former or current prostate cancer diagnosis were excluded to avoid confusing PSA testing to monitor a known diagnosis of prostate cancer with PSA screening. Those screened in the last 2 years were considered recently screened. The distribution of states from which participants answered the questions “Have you ever had a PSA test?” and “How long has it been since you had your PSA test?” was collected. A new variable to reflect clinically relevant age ranges was created, and the ages were regrouped into the ranges of 40 to 54 years, 55 to 69 years, and 70 years or older (inclusive bounds) based on US Preventive Services Task Force PSA screening guideline recommendations.^2^

### Statistical Analysis

Data were collected on November 2, 2022, and analyzed from November 2, 2022, to December 3, 2023. All data analysis was performed with R, version 4.1.3 (R Project for Statistical Computing) using core functions, the haven package for data import and manipulation, and the survey package for weighted logistic regression analyses.^5,11^ Baseline patient demographics were compared between transgender women and cisgender men using Wilcoxon rank sum, Fisher exact, and χ^2^ tests. Given the large sociodemographic disparities, transgender women and cisgender men were matched by survey year, age, race and ethnicity, annual income, employment status, educational level, and whether they reported a cost barrier to accessing health care using a 1:4 nearest neighbor propensity score matching algorithm via the MatchIt package.^11^ As current PSA screening guidelines use age as the major criteria, age was specifically included as a factor for matching.

We conducted conditional multivariable logistic regression analyses to study factors associated with recent PSA screening in the matched cohort. To assess mediating factors between recent PSA screening and gender identity, a hierarchical regression analysis was performed. A univariable logistic regression analysis was first performed to assess the association of gender identity with recent screening. We sequentially added variables (first, time since last primary care visit; second, whether a PSA test was recommended by a clinician; and third, whether a clinician-led discussion of the advantages and/or disadvantages of PSA screening occurred) into a multivariable logistic regression model while evaluating changes in the odds ratio (OR) and *P* value of the primary independent variable of gender identity, representing its association with recent screening. Within a cohort of transgender women only, we used weighted multivariable logistic regression models to measure the association of sociodemographic variables and health care access with recent screening; results were presented as ORs with 95% CIs.

Recent screening rates based on patient demographic characteristics were calculated and compared using analysis of variance tests. As recent data report potential associations among Black race, transgender female identity, and prostate cancer diagnoses, we tested the interaction between Black race and transgender women using a likelihood ratio test.^12^ As a sensitivity analysis, we also repeated key analyses using the unweighted survey data.

## Results

**Figure 1.**
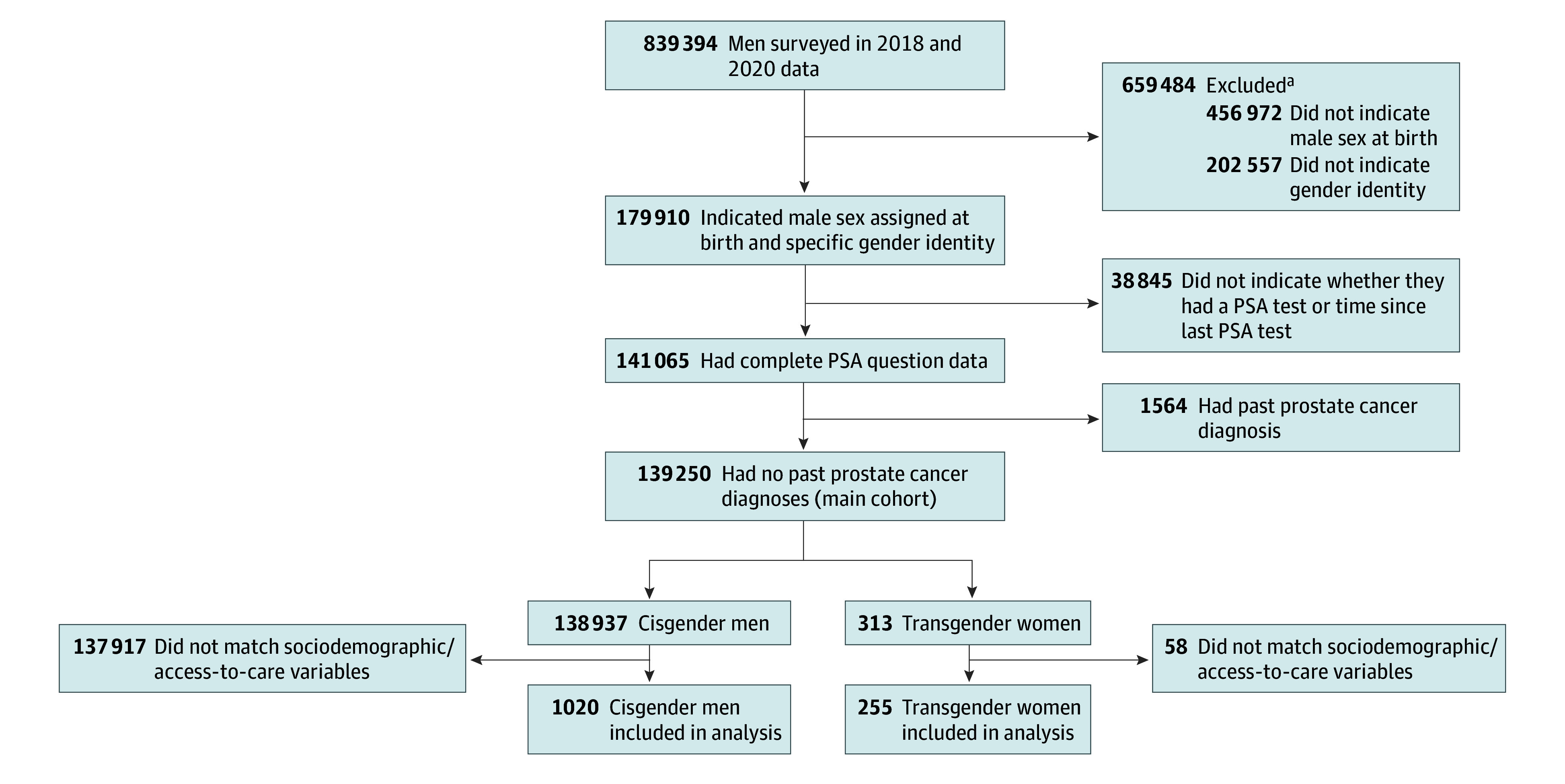
Cohort Selection PSA indicates prostate-specific antigen. ^a^Forty-five men were excluded in first round for more than 1 reason.

A total of 313 transgender women and 138 937 cisgender men met the inclusion criteria (Figure 1) and constituted a broad geographic distribution (eTable 1 in Supplement 1). Most patients in both groups were aged 55 to 69 years (50 823 cisgender men [42.7%] and 117 transgender women [45.9%]). Compared with cisgender men, fewer transgender women were non-Hispanic White (213 [68.1%] vs 111 676 [80.4%]; *P* < .001) and more transgender women were non-Hispanic Black (30 [9.6%] vs 9382 [6.8%]) and Hispanic (26 [8.3%] vs 7511 [5.4%]) (Table 1). Among those with available income data, transgender women were also significantly less likely to earn $75 000 or more annually (50 of 267 [18.7%] vs 51 790 of 122 877 [42.1%]; *P* < .001), to hold college degrees (70 of 311 [22.5%] vs 56 998 of 138 674 [41.1%]; *P* < .001), and to be currently employed (128 of 312 [41.0% vs 70 979 of 138 261 [51.3%]; *P* < .001) and more likely to report a cost barrier to health care (46 [14.9%] vs 9869 [7.1%]; *P* < .001) compared with cisgender men. Transgender women and cisgender men were matched by these sociodemographic and access-to-care variables to facilitate comparisons, which yielded a final cohort of 255 transgender women and 1020 cisgender men (570 [44.7%] aged 55-69 years). A total of 21 (1.6%) matched participants were American Indian or Alaska Native, 47 (3.7%) were Asian, 115 (9.0%) were non-Hispanic Black, 77 (6.0%) were Hispanic, 916 (71.8%) were non-Hispanic White, and 99 (7.8%) were of other race or ethnicity (which is not specified in the BRFSS).

**Table 1.** Baseline Patient Characteristics

Characteristic	Study group^a^		*P* value^b^
	Cisgender men (n = 138 937)	Transgender women (n = 313)	
Age range, y			
40-50	25 195 (18.1)	67 (21.4)	.21
51-60	33 924 (24.4)	71 (22.7)	
61-70	40 752 (29.3)	99 (31.6)	
71-80	27 731 (20.0)	60 (19.2)	
≥81	11 335 (8.2)	16 (5.1)	
Clinically relevant age group, y			
40-54	35 403 (29.8)	77 (30.2)	.40
55-69	50 823 (42.7)	117 (45.9)	
≥70	32 660 (27.5)	61 (23.9)	
Race and ethnicity			
American Indian or Alaska Native	2056 (1.5)	11 (3.5)	<.001
Asian	3396 (2.4)	13 (4.2)	
Hispanic	7511 (5.4)	26 (8.3)	
Non-Hispanic Black	9382 (6.8)	30 (9.6)	
Non-Hispanic White	111 676 (80.4)	213 (68.1)	
Other^c^	4916 (3.5)	20 (6.4)	
Annual income range, US$^d^			
0-25 000	23 637 (19.2)	115 (43.1)	<.001
25 000-50 000	26 953 (21.9)	65 (24.3)	
50 000-75 000	20 497 (16.7)	37 (13.9)	
≥75 000	51 790 (42.1)	50 (18.7)	
No. unknown	16 060	46	
Highest educational level			
No high school	9573 (6.9)	58 (18.6)	<.001
High school or GED	36 892 (26.6)	116 (37.3)	
Some college	35 211 (25.4)	67 (21.5)	
College	56 998 (41.1)	70 (22.5)	
No. unknown	263	2	
Employment status			
Employed	70 979 (51.3)	128 (41.0)	<.001
Unemployed	15 649 (11.3)	76 (24.4)	
Homemaker	346 (0.3)	2 (0.6)	
Student	251 (0.2)	3 (1.0)	
Retired	51 036 (36.9)	103 (33.0)	
No. unknown	676	1	
Last primary care visit^d^			
Past year	111 837 (81.2)	262 (84.8)	<.001
Past 1-2 y	12 366 (9.0)	17 (5.5)	
Past 2-5 y	5983 (4.3)	12 (3.9)	
>5 y	6827 (5.0)	18 (5.8)	
Never	714 (0.5)	0	
No. unknown	1210	4	
Cost barrier to care	9869 (7.1)	46 (14.9)	<.001
No. unknown	584	5	
PSA test recommended by a clinician			
Yes	61 723 (45.4)	102 (33.6)	<.001
No	74 269 (54.6)	202 (66.4)	
No. unknown	2945	9	
Survey year			
2018	70 699 (50.9)	180 (57.5)	.02
2020	68 238 (49.1)	133 (42.3)	

Abbreviations: GED, General Educational Development; PSA, prostate-specific antigen.

^a^ Includes the entire cohorts of transgender women and cisgender men prior to matching who did or did not undergo recent PSA screening. Unless otherwise indicated, data are expressed as No. (%) of patients. Percentages are calculated excluding numbers unknown. Percentages have been rounded and may not total 100.

^b^ Calculated using the Pearson χ^2^ test.

^c^ Not specified in the Behavioral Risk Factor Surveillance System.

^d^ The upper bound of the range is noninclusive.

**Figure 2.**
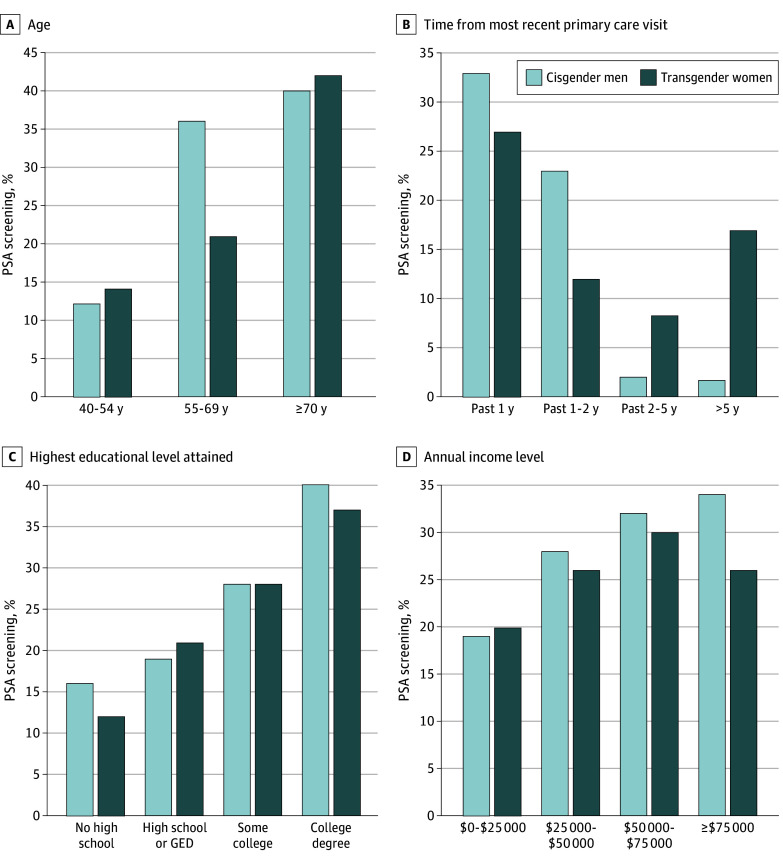
Stratified Screening Rates in Cisgender Men and Transgender Women GED indicates General Educational Development; PSA, prostate-specific antigen. Ranges for time since most recent primary care visit and income have noninclusive upper bounds.

The recent PSA screening rates were 22.2% (n = 26) for transgender women and 36.3% (n = 165) for cisgender men aged 55 to 69 years and 41.8% (n = 26) and 40.2% (n = 98), respectively, among those 70 years and older (Figure 2A). Among transgender women and cisgender men patients who had primary care visits within the last year, 273 cisgender men (33.2%) were recently screened compared with 57 transgender women (26.6%) (Figure 2B), indicating that connection to a primary care clinician alone was not correlated with recent screening. Compared with cisgender men, transgender women were less likely to report a clinician-led recommendation for PSA screening (421 [41.8%] vs 84 [32.9%]; *P* < .001). Among those who were recommended a PSA screen, recent screening rates were similar between cisgender men (257 [64.2%]) and transgender women (54 [63.4%]). Both transgender women and cisgender men experienced higher rates of recent screening with increasing levels of education (Figure 2C) and annual incomes (Figure 2D).

Transgender women had a lower likelihood of recent screening (OR, 0.65 [95% CI, 0.46-0.92]; *P* = .02) compared with cisgender men in a univariable logistic regression model assessing odds of recent screening in the cohort matched for sociodemographic and health care access variables (Table 2). In a hierarchical regression analysis, when time since last primary care visit was added, the association of gender identity with screening remained significant (OR, 0.61 [95% CI, 0.42-0.87]; *P* = .007). When the variable of whether a clinician recommended a PSA test was added, the difference in screening between transgender women and cisgender men was no longer statistically significant (OR, 0.83 [95% CI, 0.45-1.27]; *P* = .21). The results were further attenuated when accounting for clinician discussion of the advantages and/or disadvantages of PSA screening (OR, 0.87 [95% CI, 0.47-1.31]; *P* = .32). The complete multivariable model is available in eTable 2 in Supplement 1.

**Table 2.** Hierarchical Logistic Regression Analysis^a^

Variable	Association of gender identity with recent PSA screening, OR (95% CI)	*P* value
Gender identity only	0.65 (0.46-0.92)	.02
Gender identity plus time since last primary care visit	0.61 (0.42-0.87)	.007
Gender identity plus clinician recommendation for a PSA test	0.83 (0.45-1.27)	.21
Gender identity plus clinician-led discussion of PSA advantages and disadvantages	0.87 (0.47-1.31)	.32

Abbreviations: OR, odds ratio; PSA, prostate-specific antigen.

^a^ Includes the full matched cohort of transgender women (n = 255) and cisgender men (n = 1020). Variables were sequentially added into a multivariable logistic regression model while evaluating changes in the OR and *P* value of the primary independent variable of gender identity.

In a multivariable regression model in the cohort of transgender women (n = 255), patients 70 years and older had higher odds of recent screening (OR, 1.83 [95% CI, 1.02-5.49]; *P* < .001) compared with those aged 55 to 69 years (Table 3). A clinician-led PSA test recommendation had the strongest association with recent screening (OR, 12.40 [95% CI, 4.47-37.80]; *P* < .001), closely followed by the discussion of PSA advantages with a clinician (OR, 7.51 [95% CI, 2.49-24.90]; *P* < .001). Conversely, discussion of PSA disadvantages had no association with recent screening (OR, 1.14 [95% CI, 0.42-3.00]; *P* = .82). Having a college degree was associated with higher odds of recent screening (OR, 2.55 [95% CI, 1.08-14.60]; *P* = .03) compared with patients with no high school education. There was no interaction between Black race and transgender women. We repeated analyses with unweighted survey data and found similar results (eTables 3 and 4 in Supplement 1).

**Table 3.** Multivariable Logistic Regression in All Transgender Women^a^

Characteristic	OR (95% CI)	*P* value
Age group, y		
40-54	0.65 (0.17-2.37)	.52
55-69	1 [Reference]	NA
≥70	1.83 (1.02-5.49)	<.001
Race and ethnicity		
American Indian or Alaska Native	0.22 (0.01-3.11)	.30
Asian	2.97 (0.28-21.6)	.31
Hispanic	2.23 (0.21-15.9)	.43
Non-Hispanic Black	2.11 (0.45-9.98)	.33
Non-Hispanic White	1 [Reference]	NA
Other^b^	0.62 (0.08-4.33)	.62
Annual income, US$^c^		
0-25 000	1 [Reference]	NA
25 000-50 000	0.76 (0.22-2.54)	.74
50 000-75 000	1.05 (0.25-4.35)	>.91
≥75 000	0.67 (0.16-2.65)	.63
Employment status		
Employed	1 [Reference]	NA
Unemployed	1.04 (0.25-4.05)	>.92
Homemaker	0	>.92
Student	0	>.93
Retired	0.14 (0.03-1.54)	.71
Highest educational level		
No high school	1 [Reference]	NA
High school or GED	1.55 (0.33-7.88)	.60
Some college	1.99 (0.38-11.4)	.42
College	2.55 (1.08-14.60)	.03
Cost barrier to care		
Yes	1 [Reference]	NA
No	1.60 (0.36-7.61)	.52
Time since last primary care visit^c^		
Past year	1 [Reference]	NA
Past 1-2 y	0.47 (0.03-4.42)	.54
Past 2-5 y	0.40 (0.01-6.27)	.52
>5 y	0.88 (0.04-11.80)	>.90
Had a clinician recommendation for a PSA test		
Yes	12.40 (4.47-37.80)	<.001
No	1 [Reference]	NA
Had a clinician-led discussion of PSA advantages		
Yes	7.51 (2.49-24.90)	<.001
No	1 [Reference]	NA
Had a clinician-led discussion of PSA disadvantages		
Yes	1.14 (0.42-3.00)	.82
No	1 [Reference]	NA

Abbreviations: GED, General Educational Development; NA, not applicable; OR, odds ratio; PSA, prostate-specific antigen.

^a^ This multivariable logistic regression model assesses the association of the sociodemographic and access-to-care variables with the odds of recent PSA screening in the cohort of transgender women patients from the matched cohort (n = 255).

^b^ Not specified in the Behavioral Risk Factor Surveillance System.

^c^ The upper bound of the range is noninclusive.

## Discussion

In one of the largest cohorts of transgender women studied in the context of PSA screening, transgender women were less likely to undergo PSA screening compared with cisgender men matched by key sociodemographic and access-to-care variables, even when adjusting for time since primary care visits. However, this difference was progressively attenuated and no longer statistically significant when correcting for physician-led recommendations for and discussions of PSA screening. More specifically, within the cohort of transgender women, a clinician-made recommendation for a PSA test had the strongest OR associated with recent screening (OR, 0.87 [95% CI, 0.47-1.31]), suggesting that access to a clinician alone was insufficient to observe equivalent screening uptake between cisgender men and transgender women; rather, the occurrence of clinician-led discussions of the PSA test carried more weight in promoting PSA screening even when controlling for sociodemographic and health care access variables.

While the incidence of prostate cancer among transgender women is largely unknown, recent data^5^ demonstrate that the incidence of and mortality due to prostate cancer in transgender women are significant. A recent case series^12^ demonstrated a prostate cancer incidence of 14 cases per 10 000 transgender women, which is higher than in previously published data, and reported a higher incidence of aggressive disease among transgender women receiving hormone therapy due to possible diagnostic delay from the misinterpretation of PSA values in the setting of suppressive effects of estrogen. Despite the measurable risk of prostate cancer in transgender women, there are few studies on PSA screening in transgender women. A recent study among privately insured patients^13^ reported that yearly PSA screening rates between 2013 and 2019 among transgender women were lower than among cisgender men but noted that this trend narrowed when stratifying screening rates by age. They reported increasing screening rates over time and the highest PSA screening rates in transgender women older than 70 years, as similarly observed in our cohort.^13^ Other studies have reported lower rates of PSA screening and informed decision-making among transgender women compared with cisgender men and other sexual and gender minority groups.^3,14^ There is currently no consensus in the literature regarding the value of PSA screening in transgender women; a recent nonsystematic narrative review^3^ highlights the need to study the accuracy of the PSA biomarker and establish baseline PSA values in transgender women undergoing gender-affirming hormone therapy.

Our analysis highlights baseline intersectional obstacles that heighten the disconnection between health care and transgender individuals.^15^ Specifically, transgender women had lower annual income, higher unemployment rates, lower educational attainment, and higher health care cost barriers compared with cisgender men, trends that are well-described in the literature.^16,17,18,19^ Given the profound effect of these factors on comorbid conditions and health care access, we matched cisgender men and transgender women by these sociodemographic factors to minimize confounding the effects of clinician-specific interactions.^12,20^ It is well established that a significant barrier in transgender health care is lack of clinician competency, as most clinicians have little to no formal training in transgender care.^13,15^ Transgender patients have reported significant difficulty in finding knowledgeable clinicians.^21^ As a result, despite having access to care, transgender individuals may still be less likely to seek non–gender-affirming care, such as PSA screening, with the added fear of being misgendered or misunderstood by inexperienced clinicians.^21,22^ As clinician-led recommendations for PSA testing had the strongest odds associated with PSA screening in our cohort of transgender women, clinicians should take informed steps in initiating counseling in a way that is also gender affirming.

Within our cohort, transgender women and cisgender men with higher educational levels were screened at higher rates. Additionally, having a college education was associated with increased odds of recent screening in a multivariable regression model. These data are consistent with literature that establishes educational attainment as a factor that positively influences PSA screening use within the general population.^20,23^ In fact, a recent study^19^ showing lower prescreening PSA counseling rates for transgender women compared with cisgender men reported that this association was not maintained when controlling for educational level. Specific to transgender women, a Thai questionnaire study^3^ demonstrated that increased prostate cancer awareness among transgender women was associated with higher educational levels, knowledge that they retained their prostate after gender-affirming surgery, and education by the gender-affirming surgeon regarding the possibility of prostate cancer.^3^ Another study analyzing PSA screening rates using BRFSS data^24^ found these same trends persisted in underserved minority populations, as use of PSA screening increased with higher educational levels. Further, although PSA screening increased with increasing age in both transgender women and cisgender men, with those 70 years and older being recently screened at the highest frequency in transgender women (41.8%) and cisgender men (40.2%), relatively more cisgender men were screened within the recommended age range of 55 to 69 years (36.3% vs 22.2%). It is unclear whether the absence of specific guidance on screening transgender women contributed to this disparity. However, increased PSA screening among older patients (both cisgender men and transgender women) appears to be part of a larger trend recently characterized in other studies of the same BRFSS data.^25,26^

### Limitations

The limitations of our study are inherent to the nature of the BRFSS survey database. The 2018 and 2020 BRFSS surveys had response rates of 49.8% and 47.9%, respectively.^9,27^ In addition to possible selection bias, recall bias is a potential issue, as we relied on participant self-reporting. Notably, this analysis draws from national survey data, and we could not account for potential regional differences in social microclimates affecting attitudes toward transgender women that may translate to health care delivery. Our analysis also includes data from the COVID-19 pandemic, which may have affected care patterns and survey conduction. Furthermore, our cohort of transgender women was relatively small (n = 255), and almost 20% of the initial cohort of transgender women was lost in matching, which may have underpowered analyses. The use of data across 2 years also raises the possibility that some patients were interviewed twice. Data on receipt of gender-affirming hormone therapy or surgery were not available in the BRFSS 2018 and 2020 data. Given the effects of gonadotropin agonist or antagonist therapy on PSA levels, which may affect the utility of the PSA screening, knowing which patients were receiving hormone therapy could have contextualized clinician recommendations regarding screening. Transgender individuals who have access to care may also be less likely to seek cancer screening that is non–gender affirming; we cannot discern to what degree this patient-driven phenomenon affected our results.^16,21,22^ Further, the question surveying whether a clinician recommended a PSA test or discussed PSA screening advantages and/or disadvantages does not have an associated time component; these discussions could have thus taken place at any time before the PSA test. In addition, the BRFSS does not distinguish explicitly between PSA tests used for screening or monitoring of known prostate diseases.^28^ To account for this, we excluded patients with a known prostate cancer diagnosis; however, the patients who were diagnosed with prostate cancer after a routine PSA screen may have been excluded and, as there is no time component, it was not possible to determine whether the PSA screen preceded and/or initiated a prostate cancer diagnosis. The effect of a family history of prostate cancer on recent screening, which could have provided important context, could not be discerned through the BRFSS. Health literacy can also affect the efficacy of clinician-led discussions on screening^29^; however, the health literacy questionnaire was an optional BRFSS module, limiting our ability to examine this variable. Last, as these analyses were performed on a matched cohort, the differences between transgender women and cisgender men in actual practice may likely be greater than what was reported herein, as we focused this analysis on clinician variables. However, we aimed to maximize generalizability by using the CDC-provided weights in the statistical analyses.

## Conclusions

this case-control study, which to our knowledge is one of the largest cohorts of transgender women studied in the context of PSA screening, regular follow-up with a primary care clinician alone was not sufficient to observe equivalent screening uptake between cisgender men and transgender women. Rather, the occurrence of clinician-led recommendations for and discussions of the PSA screening test attenuated differences in PSA screening even when controlling for survey year, age, race and ethnicity, educational level, employment, annual income, and cost barriers to care. These data underscore the clinician’s role in influencing use of prostate cancer screening among transgender women and highlight the importance of quantifying the long-term benefits of PSA screening in transgender women.

## Notes

Supplement 1. **eTable 1.** Distribution of States From Which Participants Answered Prostate Cancer-Specific Questions Used in This Analysis **eTable 2.** Final Multivariable Model in Matched Cohort After All Variables in Hierarchical Logistic Regression Analysis Were Added **eTable 3.** Hierarchical Logistic Regression Analysis Using Unweighted Survey Data **eTable 4.** Multivariable Logistic Regression in All Transgender Women Using Unweighted Survey Data

Supplement 2.
Data Sharing Statement

## References

[1] Rawla P. Epidemiology of prostate cancer. World J Oncol. 2019;10(2):63-89. doi:10.14740/wjon1191 31068988 PMC6497009

[2] Grossman DC, Curry SJ, Owens DK, ; US Preventive Services Task Force. Screening for prostate cancer: US Preventive Services Task Force Recommendation Statement. JAMA. 2018;319(18):1901-1913. doi:10.1001/jama.2018.3710 29801017

[3] Nik-Ahd F, Jarjour A, Figueiredo J, . Prostate-specific antigen screening in transgender patients. Eur Urol. 2023;83(1):48-54. doi:10.1016/j.eururo.2022.09.007 36344317

[4] de Nie I, de Blok CJM, van der Sluis TM, . Prostate cancer incidence under androgen deprivation: nationwide cohort study in trans women receiving hormone treatment. J Clin Endocrinol Metab. 2020;105(9):e3293-e3299. doi:10.1210/clinem/dgaa412 32594155 PMC7379905

[5] Jackson SS, Han X, Mao Z, . Cancer stage, treatment, and survival among transgender patients in the United States. J Natl Cancer Inst. 2021;113(9):1221-1227. doi:10.1093/jnci/djab028 33704460 PMC8522352

[6] Sterling J, Garcia MM. Cancer screening in the transgender population: a review of current guidelines, best practices, and a proposed care model. Transl Androl Urol. 2020;9(6):2771-2785. doi:10.21037/tau-20-954 33457249 PMC7807311

[7] Centers for Disease Control and Prevention. 2020 BRFSS survey data and documentation. Updated October 27, 2022. Accessed December 3, 2023. https://www.cdc.gov/brfss/annual_data/annual_2020.html

[8] Centers for Disease Control and Prevention. Behavioral risk factor surveillance system overview. July 7, 2021. Accessed December 3, 2023. https://www.cdc.gov/brfss/annual_data/2020/pdf/overview-2020-508.pdf

[9] Centers for Disease Control and Prevention. Behavioral risk factor surveillance system 2018 summary data quality report. July 17, 2019. Accessed December 3, 2023. https://www.cdc.gov/brfss/annual_data/2018/pdf/2018-sdqr-508.pdf

[10] Centers for Disease Control and Prevention. Behavioral risk factor surveillance system comparability of data BRFSS 2020. August 6, 2021. Accessed December 3, 2023. https://www.cdc.gov/brfss/annual_data/2020/pdf/compare-2020-508.pdf

[11] Ho D, Imai K, King G, Stuart E. MatchIt: nonparametric preprocessing for parametric causal inference. J Stat Softw. 2011;42(8):1-28. doi:10.18637/jss.v042.i08

[12] Nik-Ahd F, De Hoedt A, Butler C, . Prostate cancer in transgender women in the Veterans Affairs Health System, 2000-2022. JAMA. 2023;329(21):1877-1879. doi:10.1001/jama.2023.6028 37119522 PMC10148974

[13] Premo H, Gordee A, Lee HJ, Scales CD, Moul JW, Peterson A. Disparities in prostate cancer screening for transgender women: an analysis of the MarketScan database. Urology. 2023;176:237-242. doi:10.1016/j.urology.2023.03.016 36972765 PMC10330039

[14] Ma SJ, Oladeru OT, Wang K, . Prostate cancer screening patterns among sexual and gender minority individuals. Eur Urol. 2021;79(5):588-592. doi:10.1016/j.eururo.2020.11.009 33250303

[15] Kiran T, Davie S, Singh D, . Cancer screening rates among transgender adults: cross-sectional analysis of primary care data. Can Fam Physician. 2019;65(1):e30-e37. 30674526 PMC6347308

[16] Hughto JMW, Gunn HA, Rood BA, Pantalone DW. Social and medical gender affirmation experiences are inversely associated with mental health problems in a US non-probability sample of transgender adults. Arch Sex Behav. 2020;49(7):2635-2647. doi:10.1007/s10508-020-01655-5 32215775 PMC7494544

[17] Redcay A, Bergquist K, Luquet W. On the basis of gender: a medical-legal review of barriers to healthcare for transgender and gender-expansive patients. Soc Work Public Health. 2021;36(6):615-627. doi:10.1080/19371918.2021.1942378 34340636

[18] Seelman KL, Young SR, Tesene M, Alvarez-Hernandez LR, Kattari L. A comparison of health disparities among transgender adults in Colorado (USA) by race and income. Int J Transgend. 2017;18(2):199-214. doi:10.1080/15532739.2016.1252300 33132785 PMC7597825

[19] Marthi S, O’Rourke TK Jr, Tucci C, Pareek G, Hyams E. The state of PSA counseling in male-to-female transgender patients in the US. Prostate. 2022;82(14):1315-1321. doi:10.1002/pros.24405 35748021

[20] Hararah MK, Pollack CE, Garza MA, . The relationship between education and prostate-specific antigen testing among urban African American Medicare beneficiaries. J Racial Ethn Health Disparities. 2015;2(2):176-183. doi:10.1007/s40615-014-0061-z 26863336 PMC5567979

[21] Hines DD, Laury ER, Habermann B. They just don’t get me: a qualitative analysis of transgender women’s health care experiences and clinician interactions. J Assoc Nurses AIDS Care. 2019;30(5):e82-e95. doi:10.1097/JNC.0000000000000023 31461741 PMC6738627

[22] Stroumsa D, Shires DA, Richardson CR, Jaffee KD, Woodford MR. Transphobia rather than education predicts provider knowledge of transgender health care. Med Educ. 2019;53(4):398-407. doi:10.1111/medu.13796 30666699

[23] Lerhmann-Lerche CS, Larsen SB, Andersen I, . Educational level and first-time PSA testing in general practice. Scand J Urol. 2019;53(5):275-281. doi:10.1080/21681805.2019.1681503 31663414

[24] Riviere P, Kalavacherla S, Banegas MP, . Patient perspectives of prostate cancer screening vary by race following 2018 guideline changes. Cancer. 2023;129(1):82-88. doi:10.1002/cncr.34530 36345568

[25] Kalavacherla S, Riviere P, Javier-DesLoges J, . Low-value prostate-specific antigen screening in older males. JAMA Netw Open. 2023;6(4):e237504. doi:10.1001/jamanetworkopen.2023.7504 37040113 PMC10091155

[26] Merrill RM, Otto SA, Hammond EB. Prostate-specific antigen screening according to health professional counseling and age in the United States. Prostate Cancer. 2022;2022:8646314. doi:10.1155/2022/8646314 35036010 PMC8758274

[27] Centers for Disease Control and Prevention. Behavioral Risk Factor Surveillance System 2020 summary data quality report. August 2, 2021. Accessed December 3, 2023. https://www.cdc.gov/brfss/annual_data/2020/pdf/2020-sdqr-508.pdf

[28] Bernal-Soriano MC, Parker LA, López-Garrigos M, . Factors associated with false negative and false positive results of prostate-specific antigen (PSA) and the impact on patient health: cohort study protocol. Medicine (Baltimore). 2019;98(40):e17451. doi:10.1097/MD.0000000000017451 31577771 PMC6783167

[29] Nguyen DD, Trinh QD, Cole AP, . Impact of health literacy on shared decision making for prostate-specific antigen screening in the United States. Cancer. 2021;127(2):249-256. doi:10.1002/cncr.33239 33165954

