# Supplementary material for: Prostate Cancer Screening Uptake in Transgender Women
Source: JAMA Netw Open. 2024 Feb 14;7(2):e2356088. doi: 10.1001/jamanetworkopen.2023.56088 (PMC10867675; doi:10.1001/jamanetworkopen.2023.56088)
Supplement: Supplement 1. — eTable 1. Distribution of States From Which Participants Answered Prostate Cancer-Specific Questions Used in This Analysis eTable 2. Final Multivariable Model in Matched Cohort After All Variables in Hierarchical Logistic Regression Analysis Were Added eTable 3. Hierarchical Logistic Regression Analysis Using Unweighted Survey Data eTable 4. Multivariable Logistic Regression in All Transgender Women Using Unweighted Survey Data [file jamanetwopen-e2356088-s001.pdf]

Supplementary Online Content

Kalavacherla S, Riviere P, Kalavacherla S, Anger JT, Murphy JD, Rose BS. Title.
JAMA Netw Open. 2024;7(2):e2356088. doi:10.1001/jamanetworkopen.2023.56088

eTable 1. Distribution of States From Which Participants Answered Prostate Cancer-Specific Questions Used in This Analysis

eTable 2. Final Multivariable Model in Matched Cohort After All Variables in Hierarchical Logistic Regression Analysis Were Added

eTable 3. Hierarchical Logistic Regression Analysis Using Unweighted Survey Data

eTable 4. Multivariable Logistic Regression in All Transgender Women Using Unweighted Survey Data

This supplementary material has been provided by the authors to give readers additional information about their work.

© 2024 Kalavacherla S et al. *JAMA Network Open*.

eTable 1. Distribution of States From Which Participants Answered Prostate Cancer-Specific Questions Used in This Analysis

States	Full cohort, N = 139,250[1]	All TF, N = 313[1]	Matched cohort, N = 1,275[1]	Matched TF, N = 255[1]
Alabama	0 (0%)	0 (0%)	0 (0%)	0 (0%)
Alaska	1,100 (0.8%)	3 (1.0%)	6 (0.5%)	3 (1.2%)
Arizona	0 (0%)	0 (0%)	0 (0%)	0 (0%)
Arkansas	1,394 (1.0%)	3 (1.0%)	4 (0.3%)	3 (1.2%)
California	997 (0.7%)	3 (1.0%)	3 (0.2%)	2 (0.8%)
Colorado	2,765 (2.0%)	1 (0.3%)	2 (0.2%)	1 (0.4%)
Connecticut	5,562 (4.0%)	12 (3.8%)	13 (1.0%)	7 (2.7%)
Delaware	1,282 (0.9%)	5 (1.6%)	5 (0.4%)	2 (0.8%)
District of Columbia	0 (0%)	0 (0%)	0 (0%)	0 (0%)
Florida	3,894 (2.8%)	19 (6.1%)	24 (1.9%)	18 (7.1%)
Georgia	2,257 (1.6%)	6 (1.9%)	8 (0.6%)	6 (2.4%)
Hawaii	4,640 (3.3%)	20 (6.4%)	38 (3.0%)	15 (5.9%)
Idaho	2,661 (1.9%)	4 (1.3%)	5 (0.4%)	3 (1.2%)
Illinois	2,344 (1.7%)	4 (1.3%)	6 (0.5%)	4 (1.6%)
Indiana	2,189 (1.6%)	4 (1.3%)	3 (0.2%)	3 (1.2%)
Iowa	2,769 (2.0%)	7 (2.2%)	7 (0.5%)	7 (2.7%)
Kansas	5,914 (4.2%)	9 (2.9%)	10 (0.8%)	9 (3.5%)
Kentucky	0 (0%)	0 (0%)	0 (0%)	0 (0%)
Louisiana	2,534 (1.8%)	8 (2.6%)	12 (0.9%)	6 (2.4%)
Maine	0 (0%)	0 (0%)	0 (0%)	0 (0%)
Maryland	5,094 (3.7%)	8 (2.6%)	10 (0.8%)	7 (2.7%)
Massachusetts	1,565 (1.1%)	1 (0.3%)	2 (0.2%)	1 (0.4%)
Michigan	1,836 (1.3%)	3 (1.0%)	5 (0.4%)	3 (1.2%)
Minnesota	9,241 (6.6%)	33 (11%)	37 (2.9%)	27 (11%)
Mississippi	1,581 (1.1%)	2 (0.6%)	8 (0.6%)	2 (0.8%)
Missouri	1,572 (1.1%)	1 (0.3%)	5 (0.4%)	1 (0.4%)
Montana	3,432 (2.5%)	4 (1.3%)	9 (0.7%)	4 (1.6%)
Nebraska	0 (0%)	0 (0%)	0 (0%)	0 (0%)
Nevada	863 (0.6%)	0 (0%)	0 (0%)	0 (0%)
New Hampshire	0 (0%)	0 (0%)	0 (0%)	0 (0%)
New Jersey	2,845 (2.0%)	12 (3.8%)	12 (0.9%)	7 (2.7%)
New Mexico	1,874 (1.3%)	5 (1.6%)	10 (0.8%)	5 (2.0%)
New York	13,587 (9.8%)	38 (12%)	55 (4.3%)	29 (11%)
North Carolina	2,463 (1.8%)	3 (1.0%)	10 (0.8%)	1 (0.4%)
North Dakota	0 (0%)	0 (0%)	0 (0%)	0 (0%)

© 2024 Kalavacherla S et al. *JAMA Network Open*.

Ohio	7,280 (5.2%)	15 (4.8%)	32 (2.5%)	13 (5.1%)
Oklahoma	2,613 (1.9%)	2 (0.6%)	14 (1.1%)	2 (0.8%)
Oregon	0 (0%)	0 (0%)	0 (0%)	0 (0%)
Pennsylvania	1,586 (1.1%)	4 (1.3%)	4 (0.3%)	2 (0.8%)
Rhode Island	3,019 (2.2%)	2 (0.6%)	11 (0.9%)	2 (0.8%)
South Carolina	3,882 (2.8%)	10 (3.2%)	24 (1.9%)	6 (2.4%)
South Dakota	0 (0%)	0 (0%)	0 (0%)	0 (0%)
Tennessee	1,281 (0.9%)	3 (1.0%)	12 (0.9%)	3 (1.2%)
Texas	5,160 (3.7%)	6 (1.9%)	37 (2.9%)	4 (1.6%)
Utah	2,861 (2.1%)	4 (1.3%)	7 (0.5%)	4 (1.6%)
Vermont	3,880 (2.8%)	7 (2.2%)	17 (1.3%)	6 (2.4%)
Virginia	5,104 (3.7%)	7 (2.2%)	63 (4.9%)	6 (2.4%)
Washington	7,298 (5.2%)	15 (4.8%)	79 (6.2%)	13 (5.1%)
West Virginia	3,238 (2.3%)	10 (3.2%)	162 (13%)	9 (3.5%)
Wisconsin	2,842 (2.0%)	9 (2.9%)	428 (34%)	8 (3.1%)
Wyoming	0 (0%)	0 (0%)	0 (0%)	0 (0%)
Guam	951 (0.7%)	1 (0.3%)	76 (6.0%)	1 (0.4%)
Puerto Rico	0 (0%)	0 (0%)	0 (0%)	0 (0%)

[1] n (%)

© 2024 Kalavacherla S et al. *JAMA Network Open*.

eTable 2. Final Multivariable Model in Matched Cohort After All Variables in Hierarchical Logistic Regression Analysis Were Added

**Characteristic**	**OR**[1]	**95% CI**[1]	**p-value**
**TF** (ref: CM)	0.87	0.47, 1.31	0.3
**Last primary care visit** (ref: past year)			
Past 1-2 years	0.39	0.17, 0.90	0.27
Past 2-5 years	0.34	0.09, 1.25	0.1
Over 5 years	0.52	0.15, 1.79	0.3
**Had a provider recommendation for a PSA test** (ref: No)			
Yes	14.1	7.95, 25.0	<0.001
**Had a provider-led discussion of PSA advantages** (ref: No)			
Yes	3.14	1.75, 5.66	<0.001
**Had a provider-led discussion of PSA disadvantages** (ref: No)			
Yes	1.16	0.75, 1.80	0.5

[1] OR = Odds Ratio, CI = Confidence Interval

© 2024 Kalavacherla S et al. *JAMA Network Open*.

eTable 3. Hierarchical Logistic Regression Analysis Using Unweighted Survey Data

Legend: This hierarchical logistic regression analysis measures the effect of gender identify (reference: cisgender) on the odds recent PSA screening in the full matched cohort of transgender females and cisgender males, which included 255 TF and 1,020 CM. Variables were sequentially added into a multivariable logistic regression model while evaluating changes in the odds ratio and p value of the primary independent variable of gender identity. Abbreviations: PSA = prostate-specific antigen.

Variables	Effect of gender identity (reference: cisgender) on recent PSA screening (Odds ratio [95% confidence interval])
Gender identity only	0.65 [0.43-0.96], p = 0.02
+ Time since last check up	0.61 [0.40-0.93], p=0.01
+ Provider recommendation for a PSA test	0.83 [0.63-1.24], p=0.22
+ Discussed PSA advantages with a provider<br>+ Discussed PSA disadvantages with a provider	0.87 [0.41-1.36], p=0.21

© 2024 Kalavacherla S et al. *JAMA Network Open*.

eTable 4. Multivariable Logistic Regression in All Transgender Women Using Unweighted Survey Data

Legend: This multivariable logistic regression model assesses the effects of the following sociodemographic and access to care variables on the odds of recent PSA screening in cohort of TF patients from the matched cohort (N=255). Abbreviations: ref=reference; USD = United States dollars, HS= high school; GED = general equivalency diploma, PSA=prostate-specific antigen

Characteristic	OR[1]	95% CI[1]	p-value
**Age group (ref: 55-69)**			
< 55	0.65	0.25, 1.69	0.40
≥ 70	1.2	1.06, 4.55	<0.001
**Race (ref: White (Non-Hispanic))**			
Asian (Non-Hispanic)	2.97	0.56, 15.7	0.22
Black (Non-Hispanic)	2.11	0.74, 6.05	0.24
Hispanic	2.23	0.43, 11.5	0.32
Native American (Non-Hispanic)	0.22	0.03, 1.44	0.11
Other (Non-Hispanic)	0.62	0.07, 5.14	0.70
**Income (thousand USD) (ref: 0-25)**			
25k-50k	0.76	0.24, 2.42	0.63

[1] OR = Odds Ratio, CI = Confidence Interval

© 2024 Kalavacherla S et al. *JAMA Network Open*.
