# Supplementary material for: Prostate Cancer Screening Uptake in Transgender Women
Source: JAMA Netw Open. 2024 Feb 14;7(2):e2356088. doi: 10.1001/jamanetworkopen.2023.56088 (PMC10867675; doi:10.1001/jamanetworkopen.2023.56088)
Supplement: Supplement 2. — Data Sharing Statement [file jamanetwopen-e2356088-s002.pdf]

Data Sharing Statement

Kalavacherla. Prostate Cancer Screening Uptake in Transgender Women. *JAMA Netw Open*. Published February 14, 2024. doi:10.1001/jamanetworkopen.2023.56088

Data
Data available: Yes
Data types: Deidentified participant data
How to access data: https://www.cdc.gov/brfss/data_documentation/index.htm
When available: With publication

Supporting Documents
Document types: None

Additional Information
Who can access the data: Public
Types of analyses: For any purpose
Mechanisms of data availability: Publicly available for anyone to access, without investigator support
